# Age-Dependent Changes of Adipokine and Cytokine Secretion From Rat Adipose Tissue by Endogenous and Exogenous Toll-Like Receptor Agonists

**DOI:** 10.3389/fimmu.2020.01800

**Published:** 2020-08-19

**Authors:** Verena Peek, Elena Neumann, Tomohiro Inoue, Sandy Koenig, Fabian Johannes Pflieger, Rüdiger Gerstberger, Joachim Roth, Kiyoshi Matsumura, Christoph Rummel

**Affiliations:** ^1^Institute of Veterinary Physiology and Biochemistry, Justus Liebig University Giessen, Giessen, Germany; ^2^Department of Rheumatology and Clinical Immunology, Campus Kerckhoff, Justus Liebig University Gießen, Bad Nauheim, Germany; ^3^Department of Biomedical Engineering, Osaka Institute of Technology, Osaka, Japan; ^4^Joachim Roth and Christoph Rummel, Center for Mind, Brain and Behavior (CMBB), University of Marburg and Justus Liebig University Giessen, Marburg, Germany

**Keywords:** adipokines, cytokines, batokines, lipopolysaccharide, high mobility group box-1 protein, biglycan, aging, fat explant cultures

## Abstract

White adipose tissue but recently also brown adipose tissue have emerged as endocrine organs. Age-associated obesity is accompanied by prolonged and elevated lipopolysaccharide (LPS)-induced sickness symptoms and increased cytokine and adipokine levels in the circulation partially originating from adipose tissue. In the present study, *ex vivo* fat explants were used to investigate how the exogenous pathogen-associated molecular pattern (PAMP) LPS or the endogenous danger-associated molecular patterns (DAMPs) high mobility group box-1 protein (HMGB1) and biglycan modulate the release of cytokines and adipokines/batokines and, thus, could influence systemic and/or local inflammation. The response of adipose tissue (epididymal, retroperitoneal, subcutaneous, and brown) was compared between young lean and old obese rats (2 vs. 24 months old). LPS induced a strong interleukin (IL)-6 and tumor necrosis factor (TNF) alpha release into the supernatant of all adipose tissue types investigated. HMGB1 (subcutaneous) and biglycan (retroperitoneal) led to an increased release of IL-6 and TNFalpha (HMGB1) and decreased visfatin and adiponectin (biglycan) secretion from epididymal adipose tissue (young rats). Visfatin was also decreased by HMGB1 in retroperitoneal adipose tissue of old rats. We found significantly higher leptin (all fat pads) and adiponectin (subcutaneous) levels in supernatants of adipose tissue from old compared to young rats, whereas visfatin secretion showed the opposite. The expression of the biglycan receptor Toll-like receptor (TLR) 2 as well as the LPS and HMGB1 receptors TLR4 and receptor for advanced glycation end products (RAGE) were reduced with age (TLR4/RAGE) and by stimulation with their ligands (subcutaneous). Overall, we revealed that adipokines/adipose-tissue released cytokines show some modulation of their release caused by mediators of septic (batokines) and sterile inflammation with potential implication for acute and chronic disease. Moreover, aging may increase or decrease the release of fat-derived mediators. These data show that DAMPS and LPS locally modulate cytokine secretion while only DAMPS but not LPS can locally alter adipokine secretion during inflammation.

## Introduction

The term adipose tissue actually covers several subtypes, i.e., white, brown, and beige fat. Histologically, low blood perfusion and cells with a large single lipid droplet are characteristic for white adipose tissue (WAT). Brown adipose tissue (BAT) shows multiple small lipid droplets, large amounts of mitochondria, and dense sympathetic innervation and vascularization and functions as a thermo-effector organ to generate heat during cold exposure and contributes to fever generation during infection and inflammation (1). Interestingly, deposited, structural, or fibrous fat pads (2) can be found in close vicinity to almost every organ system ranging from the kidney [perirenal fat (3)], the gut [mesenteric fat (4)], and joints [infrapatellar fat pads (5)] to the spinal cord (epidural fat) or fat surrounding blood vessels [perivascular fat (6)]. Visceral adipose tissue (VAT), for example, produces higher amounts of molecules of the innate immune system than subcutaneous adipose tissue does (SAT) (7).

Adipose tissue has a multitude of functions, including energy storage and mechanical protection (e.g., kidney). Its role as an endocrine organ emerged at least since the discovery of leptin (8)—the voice of white adipose tissue, a cytokine-like hormone, which inhibits food intake and regulates energy expenditure (9). Indeed, it is now known that adipose tissue can produce and secrete a variety of bioactive factors, including the classical pro-inflammatory cytokines interleukin (IL)-6 and tumor necrosis factor (TNF)α and the adipokines leptin, visfatin, and adiponectin (10, 11). Adiponectin increases insulin sensitivity, glucose tolerance, and fatty acid oxidation (12, 13). Visfatin regulates insulin secretion via its enzymatic action (14, 15). Visfatin, IL-6, and TNFα are primarily regarded to be pro-inflammatory, while adiponectin has some anti-inflammatory capacities (10). Visfatin has been shown to induce IL-6 and TNFα in leukocytes (16) or during adipogenic differentiation of mesenchymal stem cells *in vitro* (17) and increases circulating IL-6 *in vivo* in mice (16). Adiponectin inhibits TNFα synthesis by macrophages *in vitro* (18) or improves clinical parameters in inflammatory animal models like lipopolysaccharide (LPS)-induced liver injury in obese mice (19). Leptin rather acts as an immune modulator of the innate and adaptive immune system and, for example, contributes to the LPS-induced brain cytokine response (e.g., TNFα) in mice (20, 21). In turn, through these and other mediators, white adipose tissue plays important roles in immunity, inflammation, and acute phase responses or insulin sensitivity (22, 23).

The expression and secretion of adipokines and cytokines depends on the anatomical localization of the fat depot (24–26) and can be altered by several physiological and pathophysiological factors, which involve the balance of energy storage, hypoxia, and inflammation. Obesity (10) and age-associated obesity (27, 28) are accompanied by larger cell size of adipocytes, higher adipose tissue mass and, therefore, hypoxia, and recruitment of immune cells into the tissue causing inflammation (29–31). The microenvironment partially determines fat depot specific function and secretory activity, which is related to blood flow, cell composition, and innervation (7). Blood supply is dysregulated with aging (32), and aging also leads to fat redistribution with loss of subcutaneous and gain of visceral fat (33). Aging upregulates IL-6 and TNFα expression in mouse adipose tissue (34) while the LPS-induced release of TNFα from epididymal WAT was reported to be lower in aged than in young rats (35). Overall, obesity (36) and age-related obesity (27) lead to an imbalance of pro- and anti-inflammatory mediators cumulating in a state of low-grade inflammation (10) that has been associated with increase, for example, the risk of cognitive and emotional alterations during infection and inflammation and risk for diabetes and cardiovascular disease (23).

While endocrine function of adipose tissue is now well-established, there is increasing evidence that paracrine interaction of molecules produced and released by adipose tissue functionally alters organ systems in close vicinity (like bone or vasculature). However, the physiological significance and potential side-specific function of anatomical fat depots is still not well-understood and remains to be further investigated. As such, one third (15–30%) of circulating IL-6 is derived from fat (37). SAT was shown to significantly contribute to the systemic increase of IL-6, but TNFα rather seemed to act locally as there was no arteriovenous difference derived from a subcutaneous adipose tissue bed detected in humans (37). Almost all TNFα production and some portion of IL-6 secretion are due to tissue macrophages in white adipose tissue (38).

Here, we aimed to investigate age-dependent and fat pad-specific local release of adipokines or brown adipose tissue-derived mediators termed “batokines” (39, 40) in rats *ex vivo*. Stimulation of adipose tissue cultures with the exogenous inflammatory component and toll-like receptor (TLR) 4 ligand LPS, a pathogen-associated molecular pattern (PAMP) and mimetic of bacterial inflammation, was compared to so-called danger-associated patterns (DAMPs). These are endogenous molecules that signal “alarm” to activate the immune system, [for e.g., during chronic inflammation like high-mobility group box protein (HMGB1)] (41) or the extracellular matrix protein biglycan (42). HMGB1 is a ubiquitous nuclear protein that seems to play an important role for prolonged inflammation in aseptic (43) and septic-like LPS-induced animal models (44) or arthritis (45) when released into the extracellular space. HMGB1 signals via toll-like receptor (TLR) 4 and the receptor for advanced glycation end products (RAGE). It can, for example, be released by necrotic adipocytes, and it alters the WAT macrophage phenotype to a more pro-inflammatory state (45). Interestingly, WAT HMGB1 expression and plasma levels are higher in obese individuals (46), which seems to be primarily secreted from the stromal vascular portion (47). In addition, the extracellular matrix component biglycan could contribute to the pro-inflammatory status in WAT. Indeed, biglycan expression is increased in WAT of obese women and its expression correlates with pro-inflammatory genes such as TNFα (42). Biglycan signals via TLR2/4 and has been shown to enhance TNFα synthesis in macrophages (48).

Thus, we experimentally stimulated SAT, retroperitoneal WAT (RFAT), epididymal WAT (EFAT), and BAT with LPS, HMGB1, biglycan, or saline *in vitro* of adult (2 months, young) vs. 24-month-aged (old) rats. We used so-called fat-explant cultures, which have the advantage to preserve a more physiological cross talk between several cell types compared to cell lines, stem cell-derived cultures, or dissociated, purified cultures (49). Explants include adipocytes and the stromal vascular fraction, e.g., immune cells and endothelial cells. As the amount of BAT is very limited in adult and aged rats, we focused on LPS-induced changes for this tissue type. The age-dependent release of the cytokines IL-6 and TNFα and of the adipokines leptin, visfatin, and adiponectin was investigated. Moreover, we analyzed the expression of involved receptors for LPS, HMGB1, and biglycan, namely, TLR2/4 and RAGE, to gain some indication of a functional regulation by age and treatment.

## Materials and Methods

### Animals

WAT was withdrawn from animals killed for scientific organ removal in accordance with the German animal protection law and the local Ethics committee “Regional Council Giessen” (ethics approval numbers GI 18/2 Nr. 1/2011, GI 18/2 Nr. 485-M, 576_M, 577_M). Male young (2 months) and old (23–24 months) Wistar rats (*Rattus* sp.) originated from an in-house breeding colony with parental animals obtained from Charles River WIGA (Sulzfeld, Germany). Animals had a body weight of 200–250 g (young) or 600–750 g (old) and were housed at 22 ± 2°C, 50% humidity on a 12:12-h light-dark cycle. Rats had *ad libitum* access to water and standard lab chow with its gross composition as follows: crude protein 21.2%, crude lipids 3.8%, crude fiber 4.4%, crude ash 6.7%, calcium 1%, and phosphorus 0.7% (ssniff Spezialdiäten GmbH, Soest, Germany).

### Fat Explant Cultures

EFAT dorsal of the testicles, RFAT at the level caudal to the kidneys, inguinal SAT, and BAT derived only from the interscapular depot were dissected under aseptic conditions (70% ethanol for skin disinfection, Stockmeier GmbH & Co. KG, D-Dillenburgand, Germany), and fat explant cultures were performed as previously described (35, 50) (see also the scheme/pattern for randomization in the Supplementary Material). In brief, fat tissue was washed in cold PBS and stored on ice in Hank's Balanced Salt Solution (HBSS, Ca^2+^-, and Mg^2+^-free, Biochrom AG, D-Berlin)-filled sterile falcon tubes. Tissue was cut into 60-mg pieces, washed in HBSS, and cultured in 12-well plates with prewarmed fetal calf serum (FKS, PAA GmbH, A-Pasching, Germany) supplemented with Dulbecco's Modified Eagle Medium: Nutrient Mixture F-12 (DMEM/F12, Invitrogen, D-Darmstadt, Germany) medium at 37°C (95% humidity, 5% CO2, and 95% air) for 24 h. The next day, fat tissue was washed with FKS-free medium and incubated with 100 ng/ml LPS (*E. coli*, serotype 0111:B4, Lot: 043M4104, Sigma-Aldrich Chemie GmbH, D-München, Germany), 1 μg/ml biglycan (biglycan from bovine articular cartilage, Lot: 033M4046V, Sigma-Aldrich Chemie GmbH, D-München), 1 μg/ml HMGB1 (disulfide High-Mobility Group Box-1, LPS free (HMGB1), Lot: 170823, 18050P, 180720, 671121 HMGBiotech S.r.l., Mailand, Italy), or PBS dissolved in DMEM/F12 without FKS. BAT was stimulated with LPS [10 μg/ml] or PBS only. Supernatant was collected 24 h after stimulation and stored at −55°C for later analysis of adipokines/batokines and cytokines. LPS dose and time of incubation were chosen according to previous results showing a significant increase in IL-6, TNFα, and IL-1ra secretion into the supernatant, when compared to PBS controls (35, 50). Ten μg/ml LPS of the same serotype of LPS has previously been shown to robustly increase the secretion of cytokines from primary hypothalamic (51) and dorsal root cell cultures (52). Such a response was confirmed in preliminary dose–response experiments for BAT. Basal secretion of interleukin (IL)-6 was similar to other tissue sources indicating similar viability (Supplementary Figure 1). We selected the LPS dose that induced a similar range of response as observed in SAT for LPS-induced IL-6 secretion. Thus, 10 μg/ml LPS was chosen for treatment of BAT explant cultures. Moreover, previous studies revealed that HMGB1 and biglycan are biologically active at ~1 μg/ml concentration (53, 54). Intact cytoarchitecture was confirmed by hematoxylin and eosin stain or immunofluorescence staining of the explant tissue after culture (Supplementary Figures 2–4). Moreover, a colorimetric viability MTT assay was used to confirm that viability of all anatomical adipose tissue fat pads after dissections and before addition of PAMPs and DAMPs was similar (data not shown). As expected, the MTT assay also revealed that the metabolic activity of adipose tissue from aged rats was overall lower than from young rats (just after tissue withdrawal) and BAT had higher metabolic activity than WAT. All solution and buffers were supplemented with penicillin/streptomycin (Thermo Fisher Scientific Inc., Waltham, MA, USA) to prevent bacterial growth in case of contamination. The wet weight of fat tissue was used for normalization of mediators per ml supernatant and tissue frozen for further PCR analyses.

### Real-Time PCR

Total RNA was extracted from each sample (three 60-mg pieces of cultured fat tissue) with TRIzol (Invitrogen, Carlsbad, CA) according to the manufacturer's protocol. Reverse transcription was performed on 1 μg of total RNA in a total reaction volume of 20 μl with M-MLV reverse transcriptase (50 U), dNTP mix (10 mM), and random hexamers (50 μM; Applied Biosystems, Foster City, CA). Applied Biosystems TaqMan Gene Expression Assays and TaqMan Gene Expression Master Mix were used on a StepOnePlus Real-Time PCR System (Applied Biosystems) for relative quantification of mRNA in duplicate. Out of six reference genes tested (18S, B2M, β-actin, CANX, GAPDH, UBC; Reference Gene Assay with double-dye probe, PrimerDesign Ltd., Southampton, UK), the NormFinder software revealed β-actin as the most stable housekeeping gene for normalization of cDNA quantities. The 2^−(ΔΔCt)^ method was applied to calculate relative expression, and data is presented as relative quantity as previously reported (35, 55). The sample values represent x-fold differences from a control sample (given as a designated value of 1) within the same experiment. Analyzed genes were used with assay IDs as follows (Thermo Fisher Scientific Inc., Waltham, MA, USA): β-actin (Rn00667869_m1); HMGB1 (Rn00566331_m1); RAGE (Rn00584249_m1); TLR2 (Rn02133647_s1); and TLR4 (Rn00569848_m1). Sample numbers used for PCR are listed in Table 1.

**Table 1:** Number of samples used for PCR as well as the number of preparations from which these samples were obtained.

**Stimulation**	**Samples**	**Experiments**	**Stimulation**	**Samples**	**Experiments**
**Subcutaneous adipose tissue of young rats**			**Subcutaneous adipose tissue of old rats**		
PBS	3	3	PBS	4	4
LPS	3	3	LPS	4	4
HMGB1	3	3	HMGB1	4	4
Biglycan	3	3	Biglycan	4	4

### Measurement of Cytokines and Adipokines/Batokines

FAT-explant supernatants were analyzed for levels of IL-6 and TNFα using bioassays and adipokines/batokines including leptin, visfatin, and adiponectin using ELISA. The bioassays are based on a cytotoxic effect of TNFα on the WEHI 164 subclone 13 mouse fibrosarcoma cell line and dose-dependent growth stimulation of the B9 hybridoma cell line by IL-6 as previously reported (56). Detection limits are 3 IU IL-6/ml and 6 pg TNFα/ml. Adipokine levels were analyzed according to the manufacturer's instructions by ELISA (mouse/rat Visfatin/NAMPT RAG009R, BioVendor; mouse/rat Leptin MOB00; and rat total Adiponectin/ACrp30 Quantikine ELISA, R&D Systems). The assay ranges were 62.5–4,000 pg/ml for leptin (sensitivity 22 pg/ml), 0.5–32 ng/ml for visfatin (sensitivity 50 pg/ml), and 0.2–10 ng/ml for adiponectin (sensitivity 0.023 ng/ml). Sample numbers are listed in Table 2.

**Table 2:** Number of samples used for adipokine/batokine and cytokine measurements and the number of preparations from which they were obtained.

**Stimulation**	**Samples**	**Experiments**	**Stimulation**	**Samples**	**Experiments**
**Young rats**			**Old rats**		
**Epididymal adipose tissue (EFAT)**					
PBS	9	3	PBS	9	3
LPS	9	3	LPS	6	3
HMGB1	9	3	HMGB1	9	3
Biglycan	9	3	Biglycan	9	3
**Retroperitoneal adipose tissue (RFAT)**					
PBS	9	3	PBS	9	3
LPS	9	3	LPS	6	3
HMGB1	9	3	HMGB1	9	3
Biglycan	9	3	Biglycan	9	3
**Subcutaneous adipose tissue (SAT)**					
PBS	9	3	PBS	12	4
LPS	6	3	LPS	8	4
HMGB1	9	3	HMGB1	12	4
Biglycan	9	3	Biglycan	12	4
**Brown adipose tissue (BAT)**					
PBS	5	4	PBS	11	3
LPS	14	4	LPS	12	3

### Immunohistochemistry of TNFα and Macrophages in SAT

Adipose tissue explants were immediately snap frozen after use in culture. For immunohistochemistry, frozen tissue samples were fixed overnight in 4% formalin. Tissues were then embedded in paraffin. Five micrometer sections were prepared and deparaffinized followed by rehydration. Tissues were pretreated by boiling tissues in 10 mM citrate buffer (0.05% Tween, pH 6.0) and blocked for endogenous peroxidase activity and unspecific bindings using 5% BSA. Primary antibody (unconjugated mouse anti-human/rat TNF-α NBP2-45331 NovusBio; dilution 1:100 or AF3667 R&D Systems, goat anti-human/mouse-rat myeloperoxidase/MPO antibody) or isotype control (mouse IgM Kappa isotype MCA692 Serotec or normal goat IgG control) was incubated overnight at 4°C. Sections were incubated for 30 min with the respective Histofine detection system (Medac). For visualization, the AEC kit was used (Vector-Laboratories). Isotype control and negative control without primary antibody were negative (no red/brown signals).

### Immunofluorescence Staining of Macrophages in BAT

BAT was withdrawn from old and young rats of the interscapular depot, snap frozen in liquid nitrogen, cut into 20-μm sections (HM 500, Microm, Walldorf, Germany), thaw-mounted on poly-L-lysine-coated glass slides, and immediately used for staining. Sections were air-dried at room temperature (RT), labeled, and immersion-fixed for 10 min at RT in 2% paraformaldehyde (Merck, Darmstadt, Germany), diluted in PBS. Sections were washed three times in PBS and incubated at RT for 1 h with blocking solution [PBS, 10 % normal donkey serum (NDS; Biozol, Eching, Germany), 0.1% Triton X-100 (Sigma-Aldrich)]. The rabbit anti-MPO antibody (A0398 Dako, rabbit anti-human MPO) was used at 1:200 dilution and incubated in a humid chamber for 24 h at 4°C. After three washes in PBS, macrophages were visualized by 2-h incubation at RT with Alexa-488-conjugated anti-rabbit IgG (1:500; cat. L110A-ABP, MoBiTec GmbH, Göttingen, Germany). Glycerol/PBS solution (Citifluor, LTD, London, UK) was used as mounting medium to coverslip (glass cover slips) slides. Microscopic analysis was performed using a conventional light/fluorescent Olympus BX50 microscope (Olympus Optical, Hamburg, Germany) and a black and white Spot Insight camera (Diagnostic Instruments, Visitron Systems, Puchheim, Germany) was used to take microphotographs (MetaMorph 5.05 software). Brightness and contrast were adjusted (Adobe Photoshop 5.05). Specificity of the signals of the primary antibodies has been confirmed in previous experiments (57).

### Colorimetric Viability MTT Assay

Viability and metabolic activity of explant tissue from all anatomical fat pads of young and old rats after dissection and 24 h after culture was assessed by dimethylthiazol-diphenyl tetrazolium bromide MTT (M5655, Sigma-Aldrich) colorimetric assay.

### Data Analysis

For statistical analyses, fat explant cytokine and adipokine levels from supernatants were averaged as means (for three–four separate experiments and two–four wells/treatment and experiment) of the means ± SEM of each experimental group for statistical analysis. Relative expressions of HMGB1, RAGE, TLR2, and TLR4 of fat adipose tissue were analyzed with treatment (LPS/HMGB1/biglycan vs. control) and age (young vs. old) by two-way ANOVA (Prism 5 software; GraphPad, San Diego, CA). Bonferroni *post hoc* tests were conducted if a significant interaction was revealed. Data that was outside of an acceptable variance was excluded by outlier testing. Significance was set for *P* ≤ 5%. Data are illustrated as mean ± SEM.

## Results

### Age-Dependent LPS-Induced IL-6 and TNFα Secretion of Fat Explant Cultures

**Figure 1:**
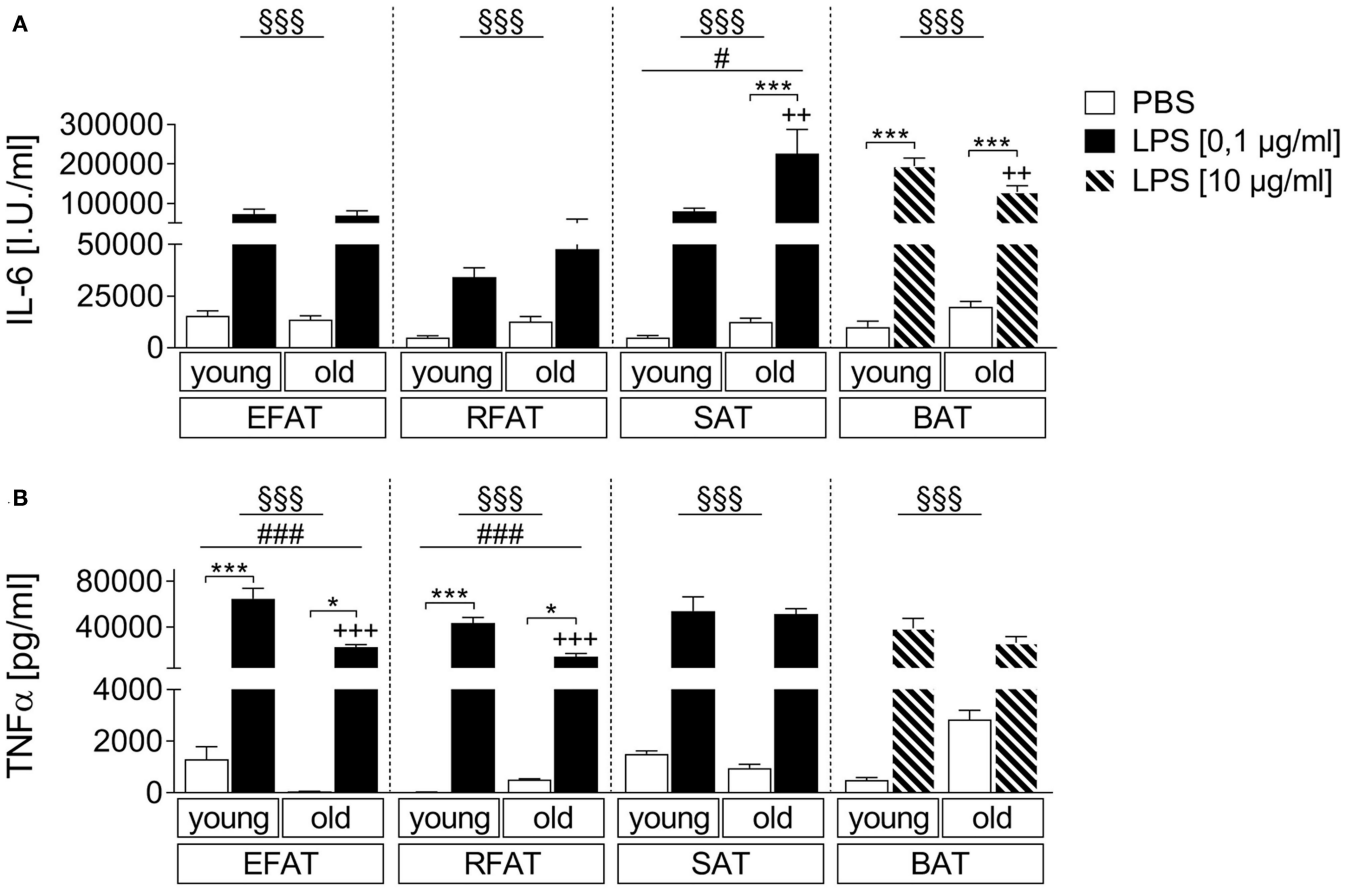
LPS-dependent changes of cytokine secretion. Increase in bioactive interleukin-6 (IL-6) **(A)** and tumor-necrosis factor-α (TNFα) **(B)** like activity in supernatants of stimulated *ex vivo* cultures. Columns represent means ± SEM. ^§^main effect treatment, ^#^main effect age, ^*^PBS vs. LPS, ^+^young vs. old; **(A)** ^§§§^*p* < 0.0001; #*p* = 0.0115; ^***^p < 0.001; ^++^*p* < 0.01; **(B)** epididymal/retroperitoneal/subcutaneous: ^§§§^*p* < 0.0001; brown: ^§§§^*p* = 0.0002; epididymal: ^*###*^*p* = 0.0002; retroperitoneal: ^*###*^*p* = 0.0001; ^*^p < 0.05; ^***^p < 0.001; ^+++^*p* < 0.001.

We compared the effects of LPS stimulation in EFAT, RFAT, SAT, and BAT on the secretion of IL-6 and TNFα 24 h after treatment compared to solvent controls. LPS increased levels of both cytokines in all different fat pads investigated (Figures 1A,B). Interestingly, IL-6 secretion was significantly enhanced in SAT while it was significantly reduced in BAT of aged rats compared to young counterparts (Figure 1A). LPS-induced TNFα secretion was significantly lower in EFAT and RFAT of old rats compared to young counterparts (Figure 1B). In addition, by means of immunohistochemistry, we demonstrated TNFα immunoreactivity in macrophages of the explants in LPS-stimulated tissue. This result shows that macrophages rather than adipocytes are the source of TNFα measured in the supernatants (Supplementary Figures 3A,B). Interestingly, the number of MPO-immunoreactive macrophages did not seem to increase but may potentially even decrease in BAT with age (Supplementary Figure 4).

### Age-Dependent HMGB1/Biglycan-Induced IL-6 and TNFα Secretion of Fat-Explant Cultures

**Figure 2:**
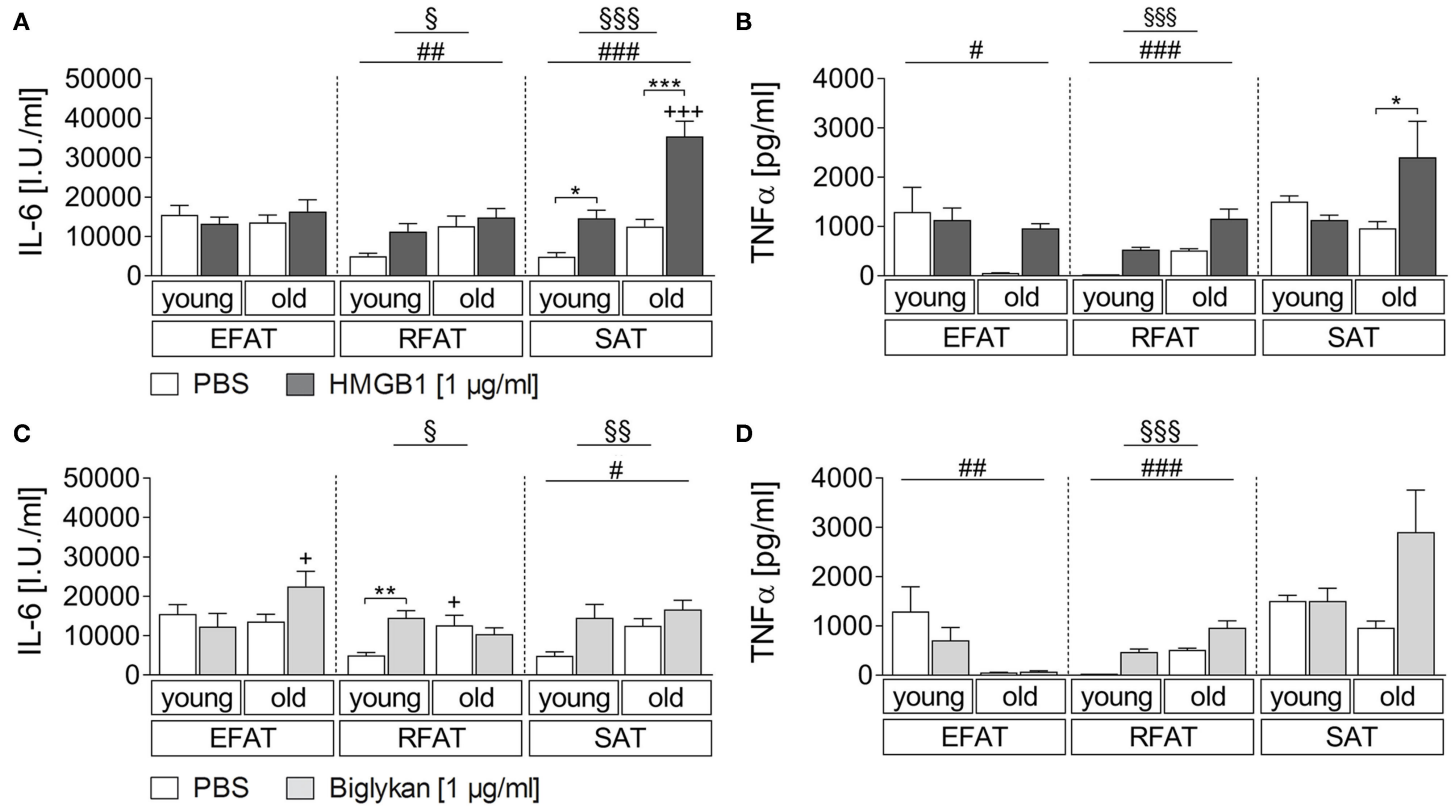
**(A,B)** Cytokine secretion of adipose tissue due to stimulation with HMGB1. HMGB1 treatment increases secretion of bioactive cytokines by retroperitoneal and subcutaneous adipose tissue. Columns represent means ± SEM. ^§^main effect treatment, ^#^ main effect age, *PBS vs. HMGB1, ^+^young vs. old; **(A)** ^§^*p* = 0.0423; ^§§§^*p* < 0.0001; ^*##*^*p* = 0.0087; ^*###*^*p* < 0.0001; ^*^p < 0.05; ^***^/^+++^*p* < 0.001; **(B)** ^§§§^*p* < 0.0001; ^#^*p* = 0.0223; ^*###*^*p* < 0.0001; ^*^p < 0.05. **(C,D)** Cytokines in supernatants in response to biglycan. There is just little modulation of adipo-cytokine secretion due to biglycan. Columns represent means ± SEM. ^§^main effect treatment, ^#^main effect age, ^*^PBS vs. biglycan, ^+^young vs. old; **(C)** ^§^*p* = 0.0482; ^§§^*p* = 0.0053; ^#^*p* = 0.0418; ^**^p < 0.01; ^+^*p* < 0.05; **(D)** ^§§§^*p* < 0.0001; ^*##*^*p* = 0.0031; ^*###*^*p* < 0.0001.

Sterile inflammatory insults commonly occur and may affect adipose tissue cytokine secretion. Thus, we applied two candidates that have been shown to play a role during sterile inflammation and to be more expressed in WAT of obese individuals (42, 45). For HMGB1, we revealed a clear fat pad-specific effect with strongest changes observed in subcutaneous adipose tissue for IL-6 and TNFα secretion (Figures 2A,B). While a main effect of age and treatment was found also for RFAT, we only detected a significant HMGB1-induced increase of IL-6/TNFα in the supernatants of explant cultures in SAT of young (IL-6) and old (IL-6 and TNFα) rats. This response was significantly enhanced by aging for IL-6 (Figure 2A), which is similar to observed changes after LPS stimulation for IL-6 but not for TNFα, suggesting some heightened inflammatory response by aging in particular in SAT. A somehow similar response appeared after biglycan stimulation (Figures 2C,D). IL-6 (RFAT and SAT)/TNFα (RFAT) levels in the supernatants showed a main effect of treatment and age. However, *post hoc* tests only revealed higher basal levels and a significant induction of IL-6 in RFAT of young rats (Figure 2C). Similar to LPS, TNFα levels were lower after biglycan stimulation in aged rats of EFAT (Figure 2D).

### Age-Dependent Secretion of Adiponectin, Leptin, and Visfatin From Fat-Explant Cultures After LPS-Stimulation

**Figure 3:**
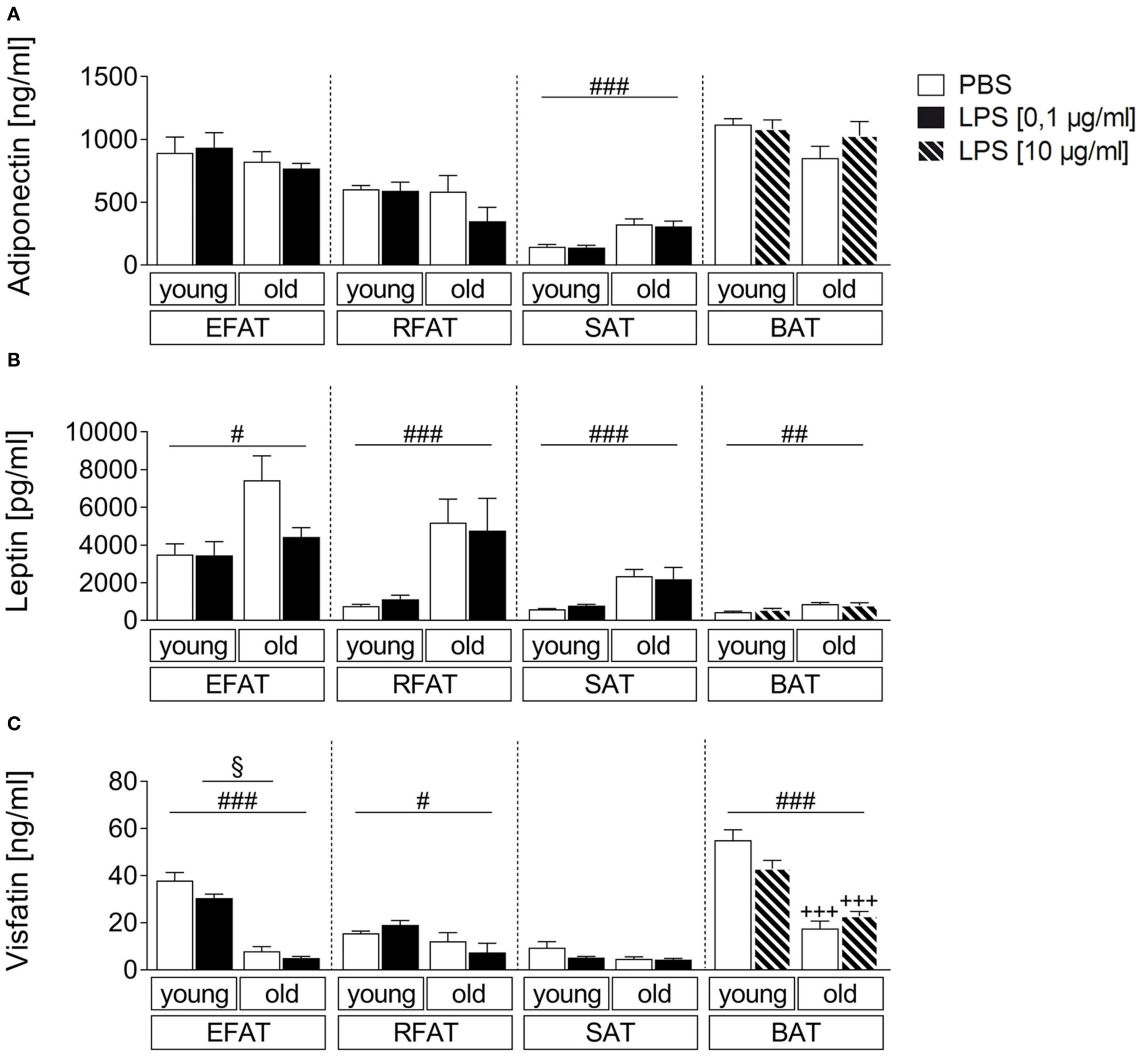
Effects of LPS on adipokine secretion. There are only small changes in adipokine secretion due to LPS inflammation. The adiponectin secretion is almost not altered by aging **(A)**. There are significantly higher leptin levels in old rats compared to young rats **(B)** whereas the visfatin concentration is significantly higher in young rats **(C)**. Columns represent means ± SEM. ^§^main effect treatment, ^#^main effect age, ^*^PBS vs. LPS, ^+^young vs. old; **(A)** ^*###*^*p* < 0.0001; **(B)** ^#^*p* = 0.0112; ^*##*^*p* = 0.0047; retroperitoneal: ^*###*^*p* = 0.0002; subcutaneous: ^*###*^*p* < 0.0001; **(C)** ^§^*p* = 0.0265; ^#^*p* = 0.0119; ^*###*^ < 0.0001; ^+++^*p* < 0.001.

In addition to classical cytokines, we aimed to investigate age-dependent secretion of the adipokines/batokines adiponectin, leptin, and visfatin in explant cultures after stimulation with the bacterial mimetic LPS. Adiponectin levels in supernatants of these cultures were higher in SAT of old rats compared with young rats (main effect of age) while its levels were not altered by age for EFAT, RFAT, and BAT (Figure 3A). Leptin secretion was higher per gram fat in supernatants of all analyzed fat pads (Figure 3B) while visfatin secretion (Figure 3C) was decreased in fat of old vs. young rats for EFAT, RFAT, and BAT (main effects of age). LPS treatment reduced visfatin levels in supernatants of EFAT (main effect of treatment) but did not significantly alter leptin or adiponectin levels in supernatants of any fat explant culture analyzed.

### Age-Dependent Secretion of Adiponectin, Leptin, and Visfatin From Fat-Explant Cultures by the DAMPs HMGB1 and Biglycan

**Figure 4:**
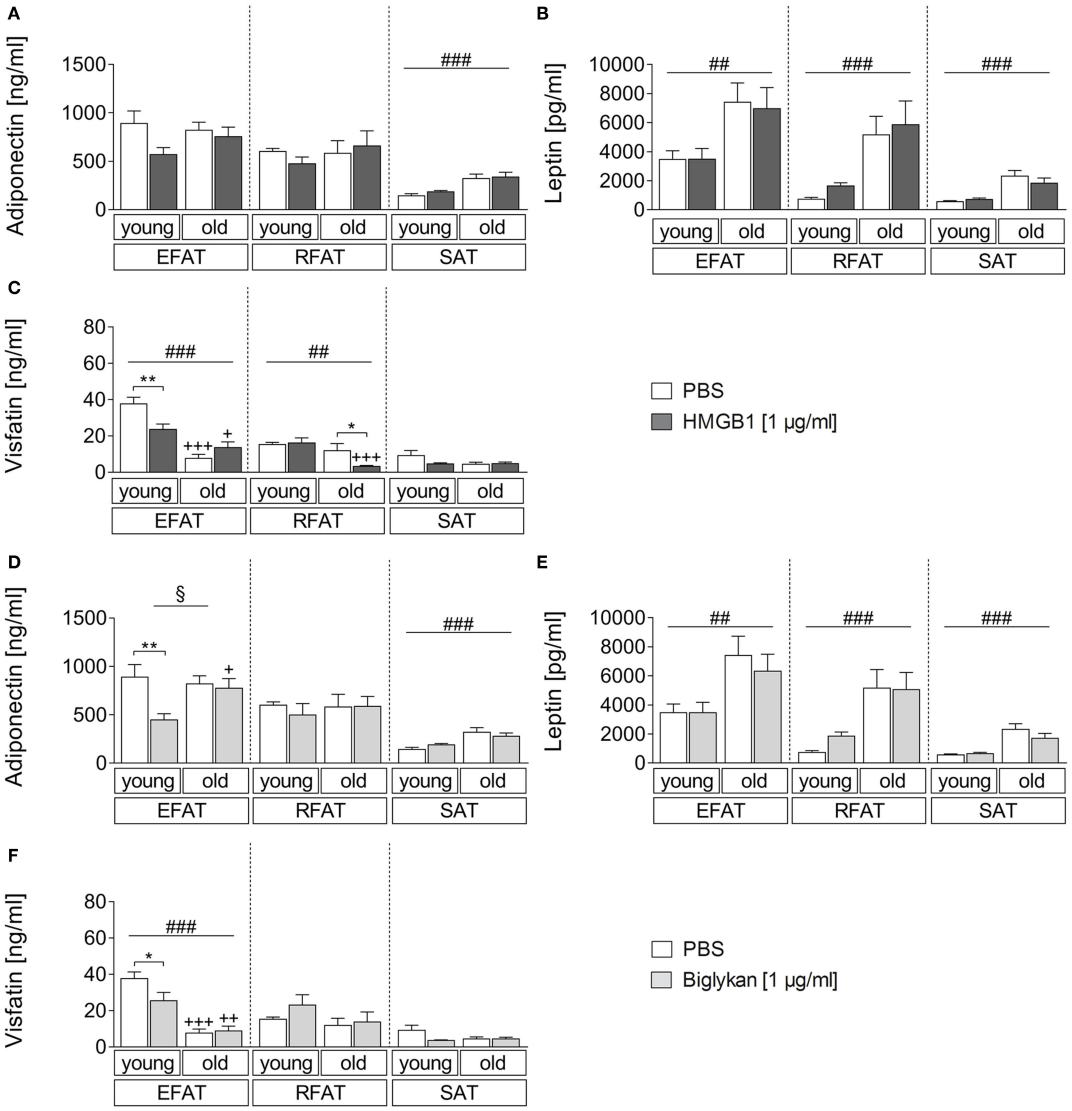
**(A–C)** Changes in adipokine secretion due to stimulation with HMGB1. HMGB1 reduced visfatin secretion in EFAT of young and RFAT of old rats **(C)**. Columns represent means ± SEM. ^§^main effect treatment, ^#^main effect age, ^*^PBS vs. HMGB1, ^+^young vs. old; **(A)** ^*###*^*p* < 0.0001; **(B)** ^*##*^*p* = 0.0015; retroperitoneal: ^*###*^*p* = 0.0002; subcutaneous: ^*###*^*p* < 0.0001; **(C)** ^*##*^*p* = 0.0014; ^*###*^*p* < 0.0001; ^*^p < 0.05; ^**^p < 0.01; ^+^*p* < 0.05; ^+++^*p* < 0.001. **(D–F)** Adipokine levels in supernatants of biglycan-stimulated *ex vivo* cultures. Adiponectin and visfatin secretion is decreased in EFAT of young rats by biglycan **(D,F)**. Columns represent means ± SEM. ^§^main effect treatment, ^#^main effect age, ^*^PBS vs. biglycan, ^+^young vs. old; **(D)** ^§^*p* = 0.0142; ^*###*^*p* < 0.0001; ^**^p < 0.01; ^+^*p* < 0.05; **(E)** ^*##*^*p* = 0.0014; ^*###*^*p* < 0.0001; **(F)** ^*###*^*p* < 0.0001; ^*^p < 0.05; ^++^*p* < 0.01; ^+++^*p* < 0.001.

Similarly, we aimed to investigate if the two DAMPs HMGB1 and biglycan can alter adipokine secretion (Figure 4). The results were comparable to those observed after LPS stimulation, confirming that adiponectin levels mainly show an effect due to age with overall higher levels in supernatants of aged rats only in SAT (Figures 4A,D). However, biglycan had a significant effect on adiponectin secretion and reduced its levels in EFAT of young but not old rats. Leptin secretion confirmed to be increased in aged rats (main effect of age) but was not altered by HMGB1 or biglycan (Figures 4B,E). For visfatin (Figures 4C,F), overall lower levels were found in WAT of aged rats (EFAT, RFAT). Interestingly, HMGB1 (EFAT, RFAT) and biglycan stimulation (EFAT) significantly reduced visfatin secretion in WAT of young (EFAT, HMGB1, and biglycan) or aged (RFAT, HMGB1) rats.

### HMGB1 and Receptor Expression for DAMPS and LPS in SAT of Young and Old Rats and Its Modulation by LPS/HMGB1/Biglycan Stimulation

**Figure 5:**
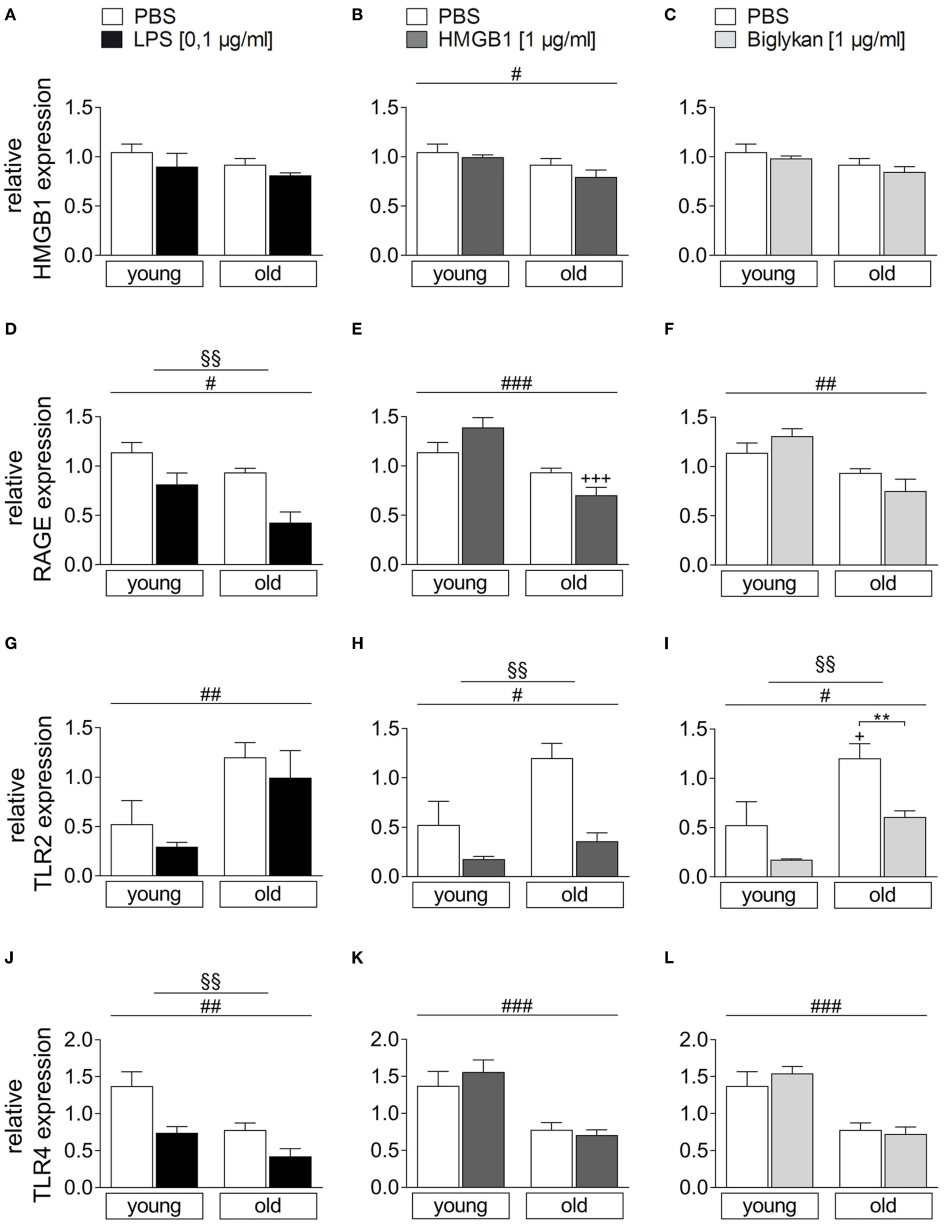
Relative expression of HMGB1 **(A–C)**, RAGE **(D–F)**, TLR2 **(G–I)**, and TLR4 **(J–L)** in the subcutaneous adipose tissue of young and old rats after stimulation with LPS, HMGB1, or biglycan compared to PBS-incubated control cultures. The relative expression is presented as the mean ± SEM. White bars show the relative expression 24 h after incubation with PBS, and the filled bars show the relative expression after incubation with LPS (black), HMGB1 (dark gray), or biglycan (light gray). The relative expression of HMGB1, RAGE, and TLR4 showed a statistically significant age-dependent main effect with a lower expression in the adipose tissue of old rats compared to the adipose tissue of young rats. In contrast, there was a significantly higher expression of TLR2 in the adipose tissue of old rats than in the adipose tissue of young rats. Incubation with LPS resulted in a significant reduction in the expression of RAGE and TLR4. Incubation with HMGB1 and biglycan caused a significant reduction in the expression of TLR2. The number of samples used for the PCR is shown in Table 1. ^§^main effect treatment; ^#^main effect age; ^*^PBS vs. TLR agonist; ^+^young vs. old; **(B)** ^§§^*p* = 0.0013; ^#^*p* = 0.0102; **(C)** ^*##*^*p* = 0.0083; **(D)** ^§§^*p* = 0.0024; ^*##*^*p* = 0.0038; **(E)** ^#^*p* = 0.0329; **(F)** ^*###*^*p* = 0.0002; ^+++^*p* < 0.001; **(G)** ^§§^*p* = 0.0037; ^#^*p* = 0.0193; **(H)** ^*###*^*p* = 0.0002; **(J)** ^*##*^*p* = 0.0022; **(K)** ^§§^*p* = 0.0012; ^#^*p* = 0.0426; ^**^p < 0.01; ^+^*p* < 0.05.

To get some insights in potential changes in receptor expression of all stimulants applied in the current study, RAGE, TLR2, and TLR4 mRNA expression was analyzed in SAT as some of the most pronounced changes for cytokines were revealed in this fat pad (Figure 5). HMGB1 mRNA expression was significantly altered neither by age nor by treatment (Figures 5A–C, only main effect of age in B). RAGE expression (Figures 5D–E) was reduced in SAT of aged rats (main effect of age) and was also reduced by LPS treatment (main effect of treatment) with some tendency for its second ligand HMGB1 (significant lower mRNA levels in aged vs. young SAT).

TLR2-mRNA expression (Figures 5G–I) was significantly higher in aged compared to young SAT (main effect of age). Moreover, its expression was significantly reduced by DAMP treatment (main effect of treatment; significant reduction for aged SAT after biglycan treatment as revealed by *post hoc* test). In contrast to these effects, the LPS receptor mRNA expression (Figures 5J–L) was reduced by treatment with its ligand, while DAMP did not alter its expression. Unlike TLR2 expression, TLR4 expression was reduced in SAT of aged rats compared to their young counterparts (main effect of age).

## Discussion

In our current study, we characterized the age and fat pad-specific potential of the exogenous PAMP LPS and the endogenous DAMPs HMGB1 and biglycan on local cytokine and adipokine/batokine secretion from RFAT, EFAT, SAT, and BAT. For the first time, the full characterization revealed that cytokine secretion can be stimulated by both exogenous and endogenous stimuli of the innate immune system, in a region-specific and comparable manner. We show enhanced LPS-induced upregulation or downregulation of IL-6 (SAT vs. BAT) or TNFα (EFAT, RFAT) secretion in fat tissue of aged vs. young rats. HMGB1 stimulated a similar response in SAT on IL-6 secretion while biglycan-induced IL-6 secretion was mainly observed in RFAT. Adipokine secretion was altered by age, i.e., increased adiponectin (SAT) and leptin (all fat pads) secretion and reduced visfatin release from all fat pads analyzed except for SAT. While LPS treatment did not influence adipokine secretion, the DAMPs HMGB1 and biglycan reduced visfatin secretion in EFAT (HMGB1 and biglycan) and RFAT (HMGB1) and adiponectin secretion in EFAT (biglycan). Interestingly, HMGB1 and LPS receptor expression (RAGE, TLR4) was significantly reduced and the biglycan receptor TLR2 was upregulated in SAT of aged rats. Stimulation with DAMPs significantly reduced the expression of their receptor in SAT of rats, suggesting some potential downregulation after their activation.

Normal plasma LPS levels can be detected around ~20–40 pg/ml in humans (58) and ~600–1,000 pg/ml in rodents (59) and increase to >1 μg/ml during experimental LPS-induced endotoxemia (e.g., 1.9 mg/kg) in mice (60) or to 10–200 ng/ml in septic human patients (60, 61). Moreover, local levels in tissue may be even higher during bacterial infection. Indeed, circulating LPS is increased in obese due to enhanced gut permeability and may alter WAT directly (59). Previous studies have also shown that adipose tissue-derived cytokines contribute to circulating cytokine levels during LPS-induced systemic inflammation (37). Moreover, fat tissue in aged obese is a significant source of circulating cytokines and adipokines during systemic inflammation (62) but can also alter tissue in close vicinity in a paracrine manner (4, 5, 63). Several previous studies have used *in vivo* systemic LPS stimulation in mice or pigs and revealed an increased expression for example of IL-6 and TNFα in adipose tissue (64–66). Others have applied fat explant cultures in cows (67), mice (68), and rats (35) and have shown LPS-induced expression and release of IL-6/TNFα. Peripheral action of LPS directly on adipose tissue using fat-explant cultures revealed that white adipose tissue of diet-induced obese rats secretes significantly higher amounts of IL-6 per g fat than from lean controls (50). Enhanced LPS-induced IL-6 expression or secretion in fat tissue after *in vivo* (62) or *ex vivo* (explant cultures) LPS stimulation, respectively, has previously been shown in aged mice compared to young counterparts and may be related to IL-1β action in aged EFAT (68). Recently, we have shown that aging-associated obese rats secret lower TNFα levels and a tendency of higher IL-6 levels per g fat than young counterparts (35). Here, we found that a significantly augmented IL-6 production from aged fat tissue is primarily present in SAT fat pads after both LPS or HMGB1 stimulation. According to the present results, some relevance for subcutaneous tissue is suggested. Interestingly, LPS-induced IL-6 expression was less increased in aged BAT compared to that of young animals.

Our present results further showed that LPS-stimulated TNFα secretion was lower in EFAT and RFAT of aged rats compared to corresponding tissue of young counterparts, confirming previous results for EFAT (35). Such an age-dependent response was less evident after HMGB1 or biglycan stimulation (main effects of age) but may be overall related to reduced functionality of resident or immigrated macrophages in the aged. Indeed, it was previously shown that almost all TNFα expression in adipose tissue arises from resident macrophages (38). Moreover, LPS-induced TNFα levels in primary human monocyte cultures were lower in aged individuals than in young adults (69). Thus, weaker LPS-induced TNFα release from EFAT and EFAT of aged compared to young rats is most likely related to some loss of secretory capacities of “aged” resident macrophages.

Interestingly, using clodronate liposomes to deplete macrophages, previous studies have shown that M1 macrophages can suppress uncoupling protein (UCP) one expression, a marker for brown adipocytes, via TNFα-mediated mechanisms (70). Moreover, numbers of infiltrating macrophages may differ between anatomical fat pads inhibiting so-called browning of white adipose tissue (71), potentially explaining some of the observed differences in the inflammatory response in fat depots to PAMPs and DAMPs. Indeed, TNFα can suppress the activity of the UCP1 promoter (72). In addition, LPS-induced inflammation previously also inhibited UCP1 expression in BAT *in vivo* (73). Using coculture experiments of brown and white adipocytes with macrophages, Dowal et al. (74) revealed that these immune cells may only enhance inflammatory response in WAT while inflammation seems to be unchanged or could potentially even be inhibited in BAT (74). However, such *in vitro* experiments will need further confirmation for their functional significance in future studies. Overall, cytokines and LPS can alter BAT-specific gene expression. Here, we have some indication that aging of BAT in rats may not be associated with increased but rather with decreased numbers of macrophages labeled with myeloperoxidase (Supplementary Figure 4). The functional significance may pertain to inhibition or modulation of inflammatory responses as discussed above but remains to be further investigated.

We and others have previously reported that circulating leptin concentrations increase during LPS-induced systemic inflammation in rodents (21, 23, 35, 75–79) and humans (80, 81), acting as a neuroimmune mediator and contributing to sickness responses like fever (35, 82) or the recruitment of neutrophil granulocytes to the brain (21, 83). However, leptin secretion seems to be primarily neuronally controlled (84). Thus, our present results that leptin levels did not increase in supernatants of fat explant cultures may be due to the fact that neuronal control is disrupted in this model system.

Like leptin, visfatin plasma levels have been reported to increase during LPS-stimulated systemic inflammation in rats (85) and humans (86) and may induce production of IL-6 and TNFα (16). Our present results confirm that release of visfatin is more pronounced from visceral adipose tissue compared to SAT (24). Interestingly, visfatin secretion was rather reduced by LPS stimulation in fat explant cultures, which might be related to other sources *in vivo* like neutrophil granulocytes that show increased levels in septic patients compared to healthy controls (87).

LPS-induced effects on the mainly anti-inflammatory adipokine adiponectin have been reported to range from reduced circulating levels in mice (64, 88), rats (89), and dogs (90) to no changes in humans (81, 91). Here, we did not find any effects of LPS on adiponectin secretion. Such differences between studies may be related to the dose and serotype of given LPS or species specifics. Interestingly, we consistently revealed that the release of adiponectin increases in SAT of old rats, which may not suffice to overall increase circulating levels with age.

In addition to white adipose tissue, brown adipose tissue is emerging as a secretory organ that may convey its beneficial effects on insulin sensitivity and metabolism not only via its thermogenic function but also by brown adipokines also termed “batokines” (39, 40). Indeed, transplantation of BAT in mice reduced body weight and ameliorates glucose homeostasis in models of diet-induced (92, 93) and genetic obesity (94). Batokines also include IL-6, which can be induced for example by stimulation with noradrenalin (39, 95). Our present results confirm that BAT stimulated with LPS releases increased amounts of IL-6 and TNFα. We are also the first to show that BAT produces high amounts of adiponectin and visfatin when compared to other fat pads. In particular, the high amounts of adiponectin secretion may actually contribute to the beneficial effects of BAT on insulin sensitivity also known for adiponectin (96). Future studies should evaluate the potential role of DAMPS in changing the secretory profile of batokines.

The DAMP HMGB1 acts as an endogenous proinflammatory mediator when released into the extracellular space (97) depending on its redox status (98). During turpentine-induced (aseptic) and LPS-induced inflammation HMGB1 plasma levels increase to 2–6 ng/ml (43) but locally most likely reach much higher concentrations. Here, we applied disulfide HMGB1, which acts similar to a pro-inflammatory cytokine. Not only is HMGB1 increased in plasma and fat tissue of obese subjects but also its inhibition by, for example, daily injection of HMGB1 antibodies (99) or genetic deletion of its receptor RAGE (100) reduced diet-induced obesity in mice. Moreover, HMGB1 may be involved in induction of the pro-inflammatory status in adipose tissue during obesity (45). For example, recombinant HMGB1 treatment of primary human adipocytes or a cell line of preadipocytes induced TLR4- (53) or RAGE-dependent IL-6 secretion (101). While our present results clearly show that HMGB1 can induce IL-6 and also TNFα secretion, this effect occurred in a fat pad-specific manner only for SAT. Thus, HMGB1 may be a more important endogenous danger signal at the barrier of the skin compared to visceral adipose tissue. In addition, we did not reveal a significant regulation of leptin and adiponectin levels in the supernatants of our cultures by HMGB1. These results are in contrast to previous findings by Montes et al. (99) who detected a significant decrease in adiponectin mRNA expression in EFAT after *in vivo* neutralization of HMGB1 in mice (99). This discrepancy could be due to the fact that mRNA expression levels do not always match with protein secretion and may also pertain to differences between *in vivo* and *ex vivo* model systems applied. Interestingly, HMGB1 stimulation significantly reduced the secretion of visfatin in EFAT of young and RFAT of old rats. Such local regulation may be regarded as a kind of local anti-inflammatory modulation. In fact, visfatin has been reported to contribute to endothelial junction disruption via HMGB1 release from endothelial cells (102), suggesting that a negative feedback mechanism on visfatin release may prevent the process from exacerbating.

Biglycan-deficient mice show reduced body weight and altered body fat composition (103). As already mentioned, its expression in adipose tissue is increased during obesity, but rather within the stromal vascular fraction than in adipocytes (42). Soluble biglycan plasma levels can be found around 200 ng/ml in healthy humans and increase [for e.g., during systemic lupus erythematosus to more than 1 μg/ml (104)]. Biglycan increases TNFα expression in macrophages, biglycan-deficient mice show reduced circulating TNFα levels after LPS-induced severe systemic inflammation (48) and its expression in adipose tissue correlates with the expression of IL-6 and TNFα (42, 105). Indeed, we show for the first time that biglycan stimulates the secretion of IL-6 in EFAT (of old rats), RFAT (of young rats), and SAT (main effect), confirming the inflammatory capacity of this endogenous alarmin. Overall, the inflammatory potential was low but may contribute to the low-grade underlying inflammation in adipose tissue during obesity. Regarding potential effects of biglycan on adipokine secretion, a previous study revealed that biglycan-deficient mice produce more adiponectin in EFAT and show higher plasma adiponectin levels (106). Our results showing that adipokine secretion was significantly reduced in EFAT of young rats are in line with this observation. Moreover, we are the first to demonstrate that biglycan can also reduce the release of visfatin from EFAT of young rats. It remains unclear why such effects by biglycan disappeared in adipose tissue of aged rats, but this observation may be related to immunosenescence of tissue resident immune cells like macrophages (107).

Overall, there exist conflicting results concerning plasma adipokine levels in aged individuals. Circulating leptin and adiponectin levels have been described to be lower (108) or higher (109, 110) in old age (111, 112). This, in turn, may be linked to variations in the definition of old age like old or very old (above 100 years), disease status, and the amount of adipose tissue (108, 109, 111). The present data on fat explant cultures in young and old rats revealed consistent increased secretion of leptin per g fat tissue in all investigated fat pads with age showing the relevance for normalization of such data toward BMI/fat mass and age. Adiponectin levels were only increased by age for SAT but not for the other fat pads investigated. We were also able to reveal that visfatin secretion is reduced in old age in visceral adipose tissue (EFAT, RFAT) and BAT. Possible underlying mechanisms are beyond the scope of this study and remain to be investigated in the future. However, we did find reduced expression of RAGE and TLR4 in SAT of old rats indicative for some immunosenescence with reduced receptor presence for LPS and HMGB1 and increased expression of the biglycan receptor TLR2.

Concerning the cellular source of cytokines and adipokines, previous studies have used several isolation and separation protocols revealing that the stromal vascular fraction and adipocytes differentially contribute to the total source of mediators secreted from adipose tissue. All mediators investigated here except TNFα, which is primarily produced by tissue macrophages (38), have been shown to be produced by adipocytes (113). Moreover, visfatin is also produced by macrophages or other cell types and IL-6 as well by stromal vascular fractions cells (113). Brown adipocytes secrete batokines like adiponectin or the cytokine IL-6; less is known about the respective contribution of other cells of the stromal vascular fraction to the secretion of mediators from brown adipose tissue (39, 114). While the preparation of BAT was performed in the very specific interscapular localization of visually brown adipose tissue for fat explant cultures in the current study, some minor portion of beige or white adipose tissue may have contributed to secreted mediators. Thus, the exact cellular source in BAT explant cultures remains to be investigated in the future, e.g., for leptin and visfatin. Interestingly, using immunohistochemistry for TNFα or myeloperoxidase (MPO, macrophages), we obtained evidence that macrophages indeed represent a central source of TNFα production within SAT (Supplementary Figure 3). In BAT, macrophage numbers did not seem to increase but may even decrease with age as revealed by immunofluorescence MPO staining (Supplementary Figure 4), suggesting that other cell types may also contribute to the source of TNFα in this type of tissue.

It has already been shown that the expression and secretion of adipokines are dependent on fat pad-specific localization (24–26). Mesenteric adipose tissue may be protective for intestinal inflammation, for example, by shaping a more anti-inflammatory macrophage phenotype (M2) (4, 63). Infrapatellar fat pads have also been shown to contribute to production of adipokines and cytokines during inflammatory conditions like osteoarthritis (5), and we and others have shown that adipokines modulate osteoarthritis and bone remodeling (11, 115). Moreover, the systemic release of these mediators during obesity modulates inflammatory insults for example in the brain; thus, some of them seem to have therapeutic potential (10, 23, 116).

Overall, LPS strongly increased cytokine secretion but adipokine/batokine levels were not significantly affected, while DAMPs only moderately altered cytokine secretion but significantly reduced adiponectin and visfatin secretion almost exclusively in young epididymal fat pads. The expression of receptors for the exogenous PAMP LPS, namely, TLR4 (45) or RAGE (117), were reduced after LPS or HMGB1 treatment in the explant cultures, suggesting their downregulation, which is known to occur to induce tolerance effects (118). Moreover, TLR2 expression was primarily reduced by biglycan stimulation, which is in line with its signaling cascade activation.

In summary, we revealed that the endogenous DAMPs HMGB1 and biglycan can locally impact adipokine and cytokine secretion supporting the hypothesis that these mediators may be important modulators of adipose tissue inflammation that occurs during diet- or age-related obesity. Moreover, we expand on the emerging role of batokines like adiponectin, which may contribute to beneficial effects of this metabolically highly active tissue type. In the future, more insights on fat pad-specific local paracrine interactions with a multitude of organ systems will help to better understand fine-tuning of organ function by adipose tissue.

## Limitations

Limitations of fat explant cultures pertain to the fact that only short-term cultures are meaningful, and the explant size is limited for appropriate energy and oxygen supply and the lack of functional innervation. Differential sympathetic innervation of anatomical fat pads may be needed for fine-tuning of side-specific function (119). Thus, more *in vivo* analyses will be necessary using, [for e.g., microperfusion of different fat pads to gain complementary insights into the local production of adipokines/batokines and cytokines (120)]. In addition, our current findings cannot be assigned to functional contributions of primary cultures of a defined cellular phenotype when using cultures of single-cell types. Nonetheless, previous studies support the assumption that macrophages play an important role for the inflammatory response of this type of tissue. Indeed, accumulation of macrophages in WAT is a hallmark of the low-grade inflammatory state during obesity (10, 23). Moreover, viability of bouts of tissue, which may differ between sources and age, is an important factor to consider when comparing different anatomical fat pads or brown and white tissue. Therefore, we have separately analyzed all types of adipose tissue as we are aware that due to blood supply or the local milieu bouts of tissue may have different properties. To prevent any local effects within one anatomical localization, tissue bouts were randomized and mixed from cranial, middle, and caudal parts of the tissue as previously reported (50) (see also the scheme/pattern for randomization in the Supplementary Materials). Here, we also show evidence for basal levels of IL-6 in all types of adipose tissue investigated in a very small and similar range, only revealing some slight differences suggesting that viability was overall similar as well. Indeed, IL-6 is produced by *de novo* synthesis, which prerequisites viable cells for productions and release into the supernatants (Supplementary Figure 1). In addition, intact cytoarchitecture is confirmed by hematoxylin and eosin stain of the explant tissue after culture (Supplementary Figure 2) and a vitality test using the colorimetric MTT assay confirmed the overall similar viability of anatomical fat pads (data not shown). Within the scope of the present manuscript, we did not investigate the functional significance of cytokine secretion from adipose tissue. Future studies will be needed to dissect out their contribution to changes in adipokine/batokine production. While we confirm that TNFα is mainly present in macrophages within the fat explants representing a crucial cell type for cytokine production (Supplementary Figure 3), we did not reveal any correlation of TNFα levels in supernatants with adipokine secretion, suggesting that it may have a limited role in paracrine modulation of this response (Supplementary Figure 5). Similarly, IL-6 did not or only very marginally correlate with adipokine levels in the supernatants, which does not suggest any biologically significant paracrine role for IL-6-mediated modulation of adipokine secretion.

## Data Availability Statement

The raw data supporting the conclusions of this article will be made available by the authors, without undue reservation.

## Ethics Statement

The animal study was reviewed and approved by Ethics committee Regional Council Giessen, ethics approval numbers GI 18/2 Nr. 1/2011, GI 18/2 Nr. 485-M, 576_M, 577_M.

## Author Contributions

EN, JR, KM, and CR contributed to the conception and design of the study. *Ex vivo* experiments and sample preparation were conducted by VP, FP, SK, TI, JR, and CR. VP performed the statistical analyses. Data analyses and interpretation were done by VP, EN, and CR. VP, EN, JR, FP, RG, SK, TI, KM, and CR contributed to writing the article and to revising the content. All authors proofread the final manuscript.

## Conflict of Interest

The authors declare that the research was conducted in the absence of any commercial or financial relationships that could be construed as a potential conflict of interest.
