# Supplementary material for: Age-Dependent Changes of Adipokine and Cytokine Secretion From Rat Adipose Tissue by Endogenous and Exogenous Toll-Like Receptor Agonists
Source: Front Immunol. 2020 Aug 19;11:1800. doi: 10.3389/fimmu.2020.01800 (PMC7466552; doi:10.3389/fimmu.2020.01800)
Supplement: Supplementary file 1 [file Data_Sheet_1.PDF]

Supplementary material

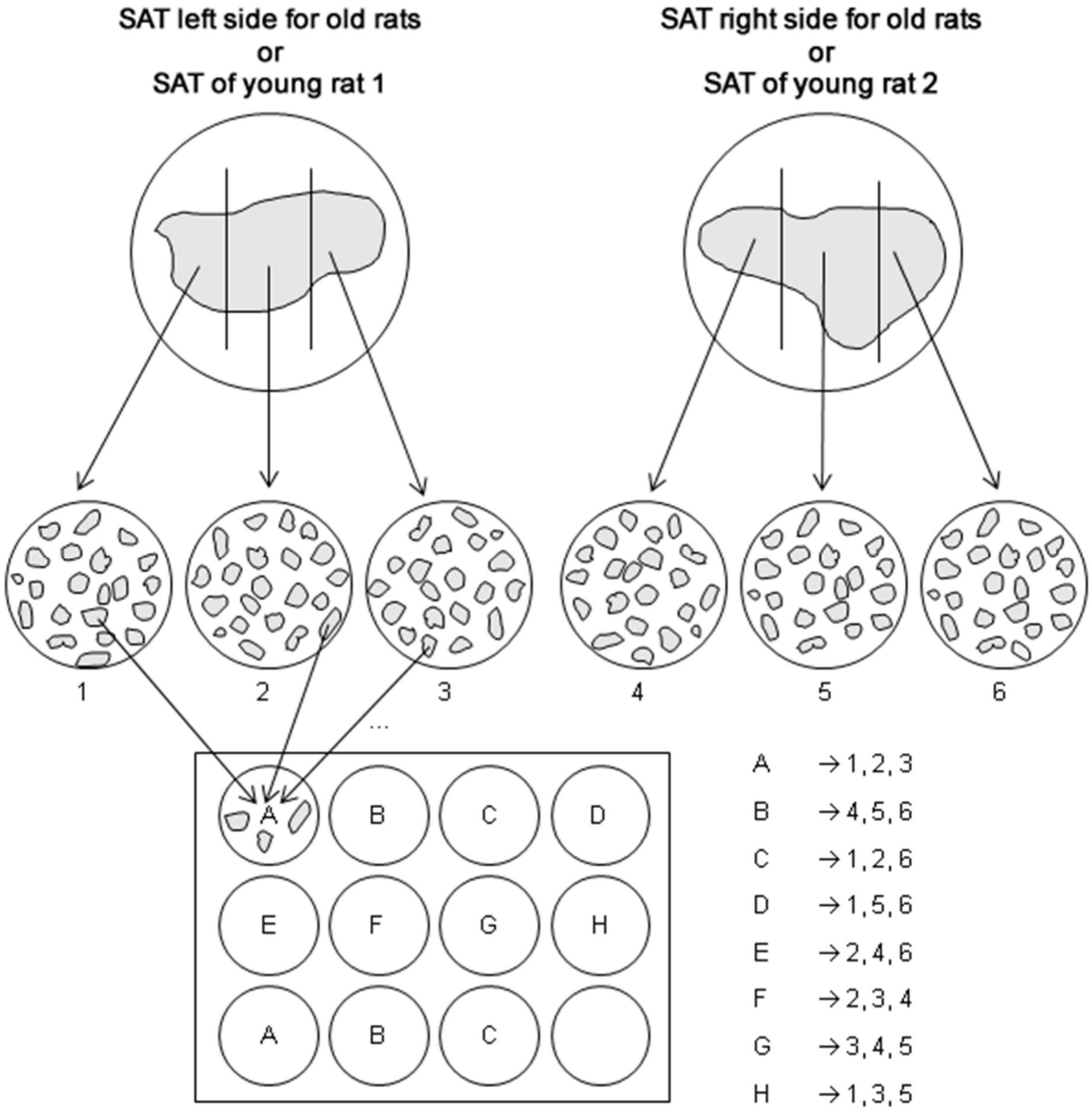

A representative scheme/pattern for randomization is shown for one single experiment. White adipose tissue of two young rats and one old rat was used for one explant experiment.

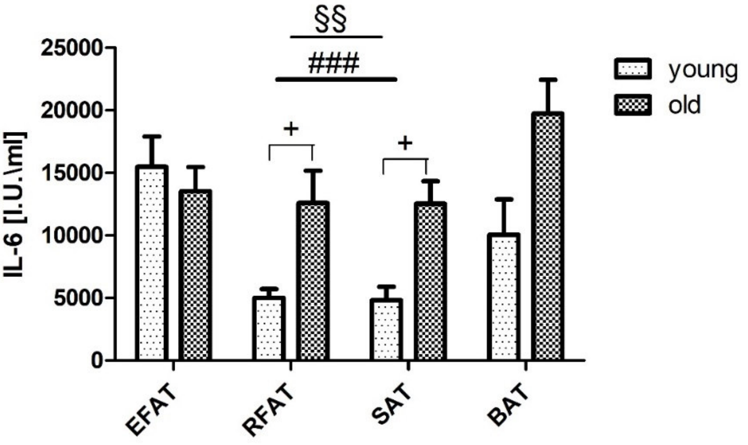

Supplementary Fig. 1: Basal IL-6 level in supernatants of fat explant cultures 24 hours after stimulation with PBS in all anatomical fat pads investigated. Overall, a significant main effect was detected for differences by source and age. With significant interaction, Bonferroni multiple comparisons revealed significant higher basal IL-6 levels in supernatants in aged RFAT and SAT compared to young counterparts. Thus, some differences in IL-6 producing capacity may have contributed to observed changes while these effects do not seem to translate into observed robust inducible production and release of IL-6 in explant cultures after LPS- or DAMP-stimulation. A similar magnitude of IL-6-induction was observed between sources of adipose tissue and age of the animals. Overall, we believe that our present data still provides useful information to describe age-dependent differences for several sources of fat pads. Columns represent means ± SEM. § main effect of source, # main effect age, + young vs. old; (A) §§ $p < 0.05$; ### $p < 0.0001$; + $p < 0.05$;

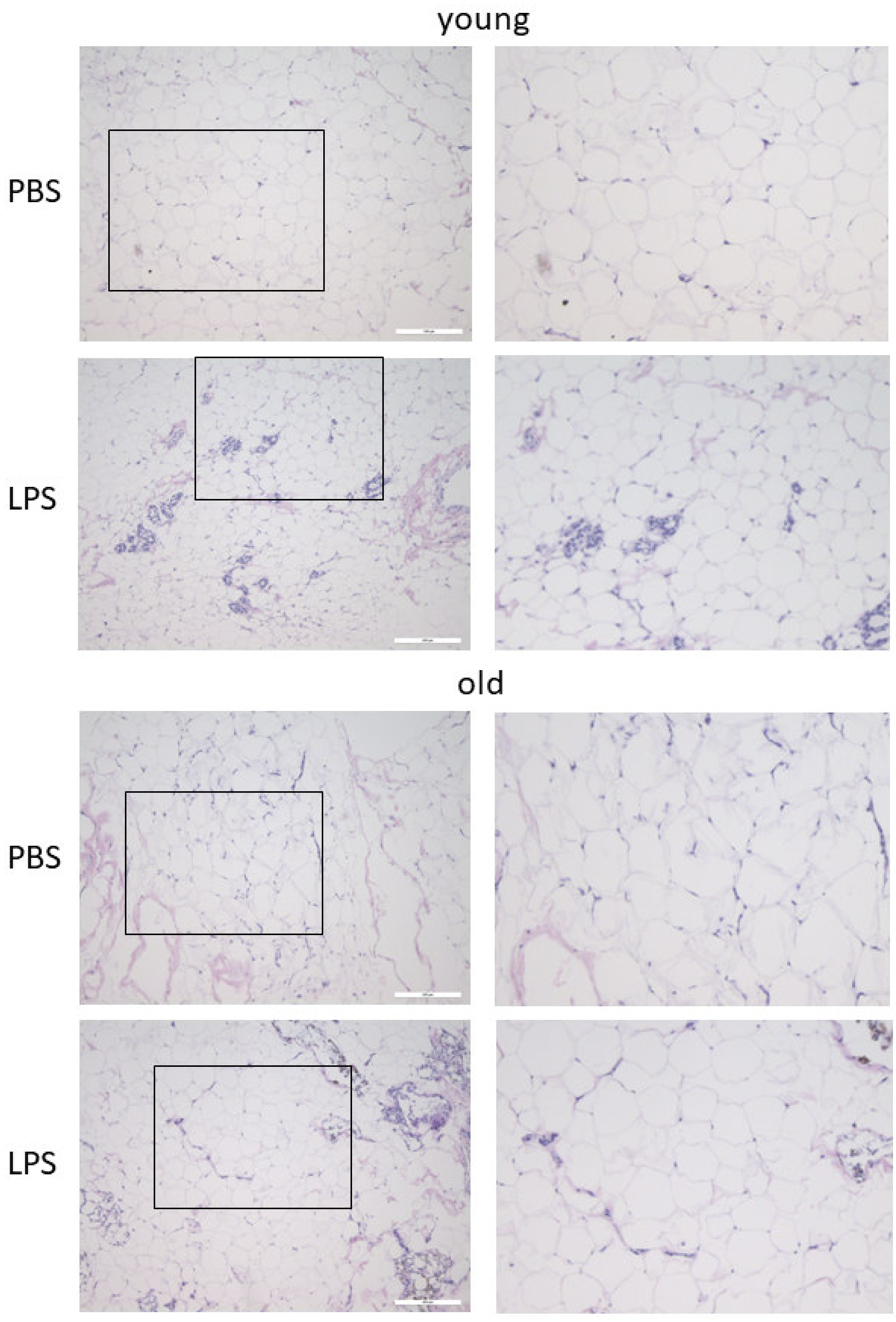

Supplementary Fig. 2: Intact cytoarchitecture is confirmed by haematoxylin and eosin (H/E) staining of subcutaneous adipose tissue (SAT) explants after culture and stimulation with LPS or PBS for 24 hours; scale bars represent 200 µm

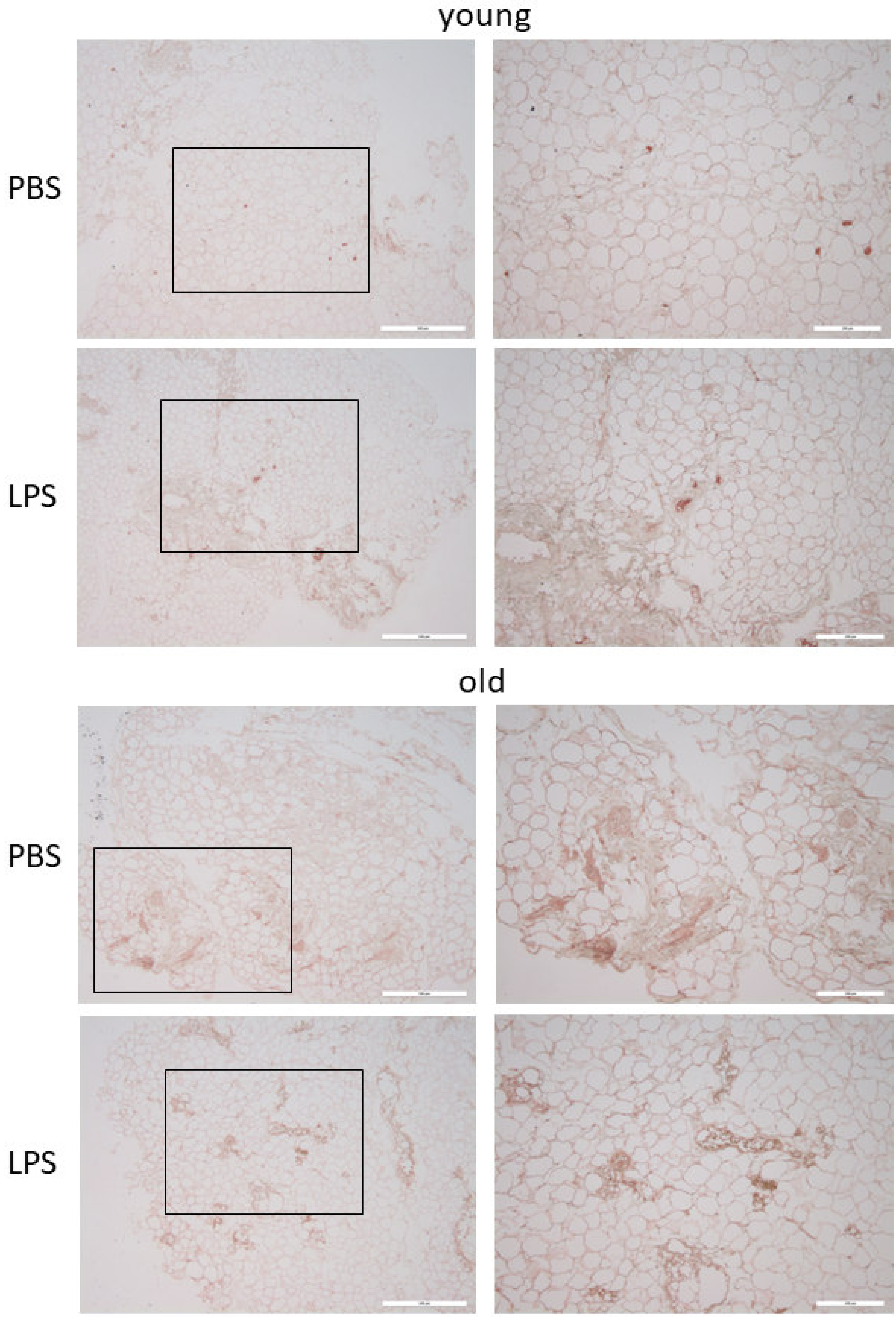

Supplementary Fig. 3A: Immunohistochemistry for TNFα shows increased signals for TNF (red/brown) in the LPS treated animals in both, young as well as aged rats of subcutaneous adipose tissue (SAT) explants after culture compared to PBS-treatment 24 hours after stimulation; scale bars represent 500 µm (left) or 200µm (right), respectively; representative microphotographs are shown out of two independent experiments.

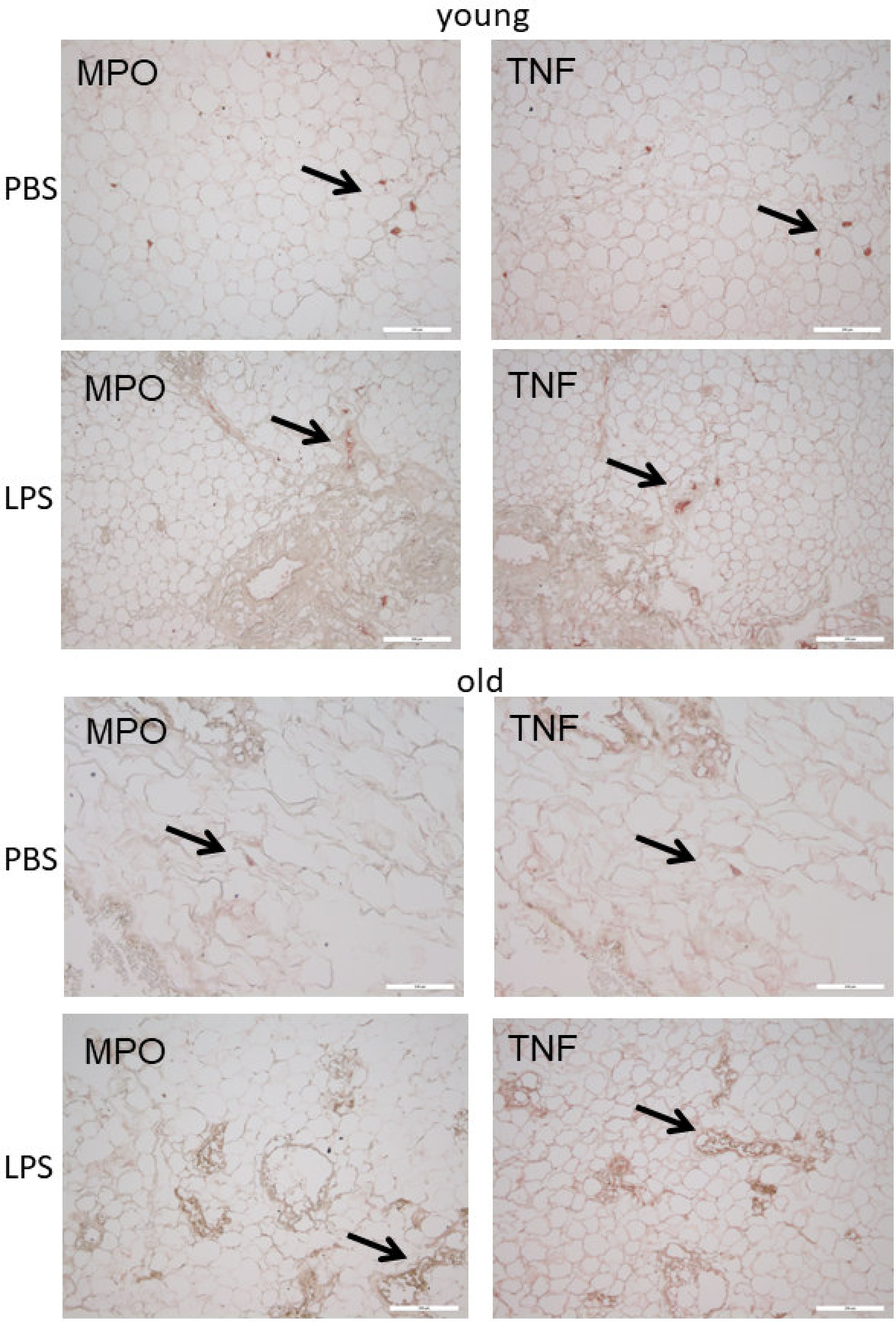

Supplementary Fig. 3B: Immunohistochemistry for myeloperoxidase positive macrophages (red/brown) reveals that TNFα immunoreactivity (red/brown) is present in these cell types in both, young as well as aged rats of subcutaneous adipose tissue (SAT) explants after culture stimulated with LPS compared to PBS-treatment 24 hours after treatment. Please note that the corresponding sections PBS old MPO/TNF (see also

Figure 3A) were replaced with microphotographs at higher magnification as these tissue sections almost all detached form the slides during the MPO staining process; scale bars represent 200 µm, 100µm for PBS old (MPO/TNF). Manual counting of macrophage numbers in two sections per animal (two to four animals per group out of two independent experiments) revealed a trend for higher numbers of macrophages with age (data not shown). Statistical evaluation was not performed due to low numbers of biological replicates.

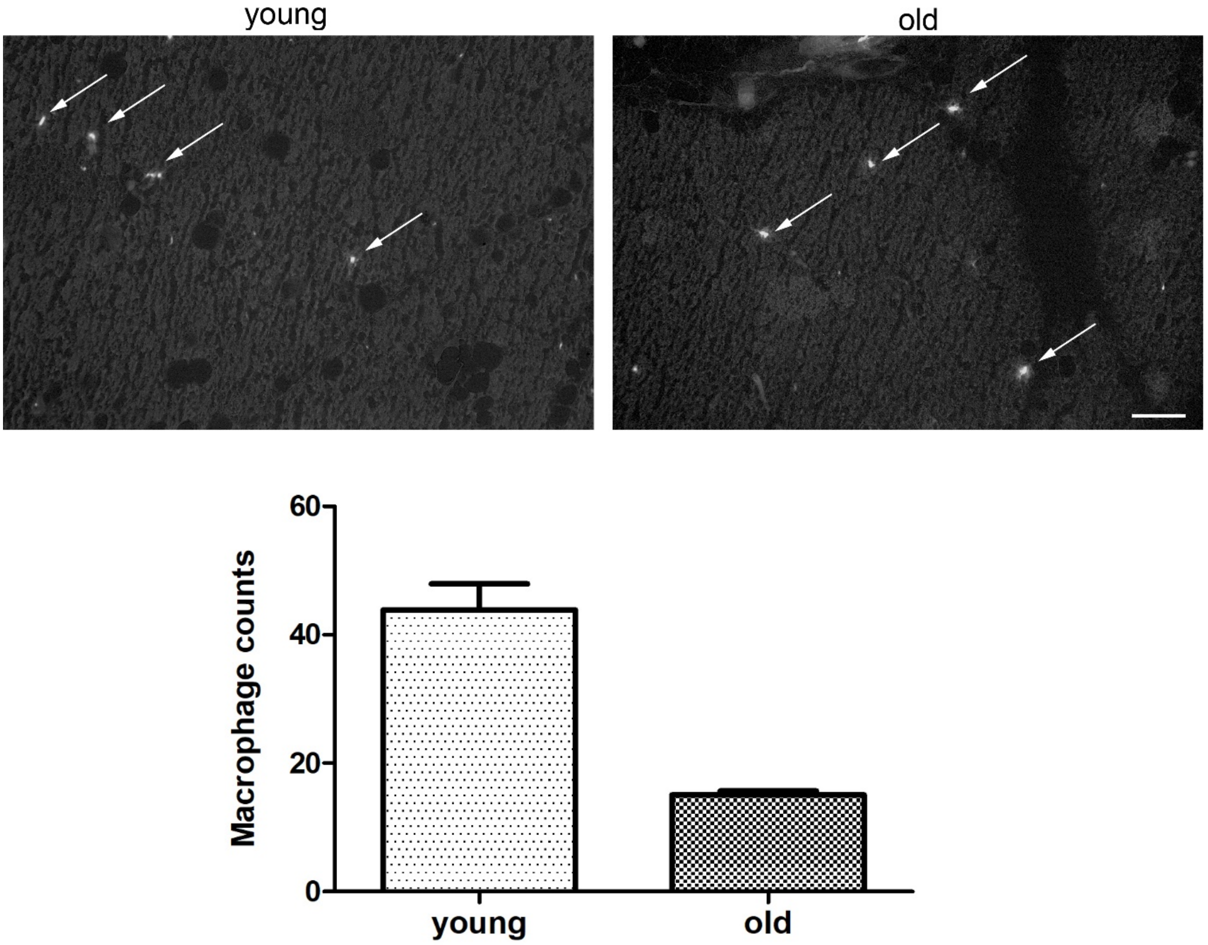

Supplementary Fig. 4: The numbers of myeloperoxidase stained macrophages (white arrows) do not increase but may even decrease in BAT of old compared to young rats; scale bar represents 100µm; microphotographs of three fields of view per animals were analyzed using the Metamorph automatic counting tool. Means for each animal are displayed as mean per group (young versus old). Freshly isolated BAT from four young and two old animals was used for staining (biological replicates). Due to low numbers of biological replicates no statistical analyses is performed.

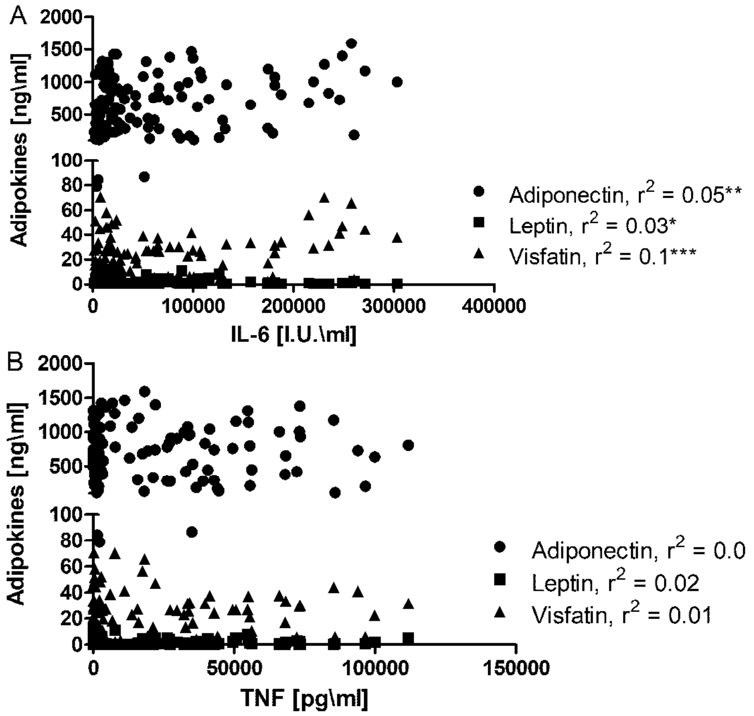

Supplementary Fig. 5: Secreted cytokines do not correlate with adipokines in supernatants of explant cultures (A) IL-6 levels in supernatants show some significant correlation with adipokines, however, did not reach any biologically relevant positive coefficient of correlation (pearson) 24 hours after LPS or PBS-stimulation of all explant cultures pooled (EFAT, RFAT, SAT, BAT). (B) TNFα did not at all correlate with adipokines in supernatant of these cultures.
